# A mixed methods study of employers’ and employees’ evaluations of job seekers with a mental illness, disability, or of a cultural minority

**DOI:** 10.3233/WOR-213568

**Published:** 2021-09-28

**Authors:** Tonje Fyhn, Vigdis Sveinsdottir, Silje E. Reme, Gro M. Sandal

**Affiliations:** a NORCE Research Health, Bergen, Norway; b Department of Psychology, University of Oslo, Oslo, Norway; c Faculty of Psychology, University of Bergen, Bergen, Norway

**Keywords:** Diversity, equal opportunities, selection, evaluation, work participation

## Abstract

**BACKGROUND:**

Groups in society that are under-represented in the workforce encounter various barriers in the job-seeking process. Some of these barriers are found on the employer’s side of the table.

**OBJECTIVE:**

This study investigates supervisors’ and employees’ assessments of job seekers with different forms of disabilities, health issues, or with a minority background. It also investigates respondents’ previous experience with such colleagues, and whether supervisor status affects their assessments.

**METHODS:**

A survey was distributed among supervisors (*n* = 305) and employees (*n* = 925) using a vignette design with ten characters, inquiring about willingness to include such an employee in their work group. The vignettes described job seekers with either a mental illness, a physical disability or a cultural minority. Risk ratio (RR) was calculated for being assessed positively, using a vignette character describing a single mother as reference.

**RESULTS:**

Vignette characters describing mental health issues and physical disabilities were less likely to be assessed positively than the reference case, except for the vignette describing audio impairment. Cultural minorities were assessed as positive, or more positively than the reference case. Supervisors and employees generally agreed in their assessments of vignette characters, and previous experience was consistently associated with a more positive assessment of the character in question. Various barriers to include the least favoured vignette characters were identified.

**CONCLUSIONS:**

Although some findings are promising with regard to increasing work participation for underrepresented groups, barriers pertaining to some of the vignette characters should be addressed in vocational rehabilitation efforts, as well as in organizations seeking to enhance equal opportunities and diversity.

## 1 Introduction

Efforts to increase work participation among groups that are underrepresented in the workforce are important for several reasons. Many societies face an ageing population, and utilizing a larger portion of the work-capable population is crucial to sustain economic growth and welfare services. From the individual perspective, work participation for cultural minorities and people with disabilities has become not only a civil rights issue, but also a health issue, as the positive association between work and health has become well documented [1, 2]. In spite of increased cultural diversity in society, and advances in physical accessibility and technical aids, the work force does not mirror the ethnic and functional diversity in the general population. The employer is a critical gatekeeper of employment, and understanding their considerations in the hiring process can identify barriers salient for certain groups.

### 1.1 Previous studies

Previous studies have found that employers prefer to hire persons with a physical disability over someone with mental health issues [3–5], but always prefer employees without a health issue [6, 7]. Interview studies have revealed that perceived risk factors in employers’ assessment of applicants with a disability include concerns with productivity level, skills set, fit with work environment, absenteeism and uncertainty of accommodation needs [8–10]. One study found that employers’ specific concerns varied between employees with physical disabilities as opposed to mental health issues [11]. Mental health conditions were generally regarded as more diffuse and challenging to handle than physical disabilities. For job applicants representing a cultural minority, experimental studies have found that those who appear integrated in the majority culture are favoured among employers [12], and that job seekers with an ethnic name get fewer call-backs than job seekers representing the majority culture [13–15].

Studies investigating the influence of having previous experience with working together with people with disabilities have shown mixed results: While some studies show an association between experience and positive attitudes towards people with disabilities [16], other studies have failed to establish the same association [17]. Unger [17] found that studies conducted before the Americans with Disabilities Act, found that experience was associated with more positive attitudes; however, studies conducted after the act was passed indicated that employers expressed positive attitudes irrespective of experience. This may indicate that the effect of experience is hard to establish due to social desirability.

In the current study, Stone and Colella’s model of “Factors affecting the treatment of disabled individuals in organizations” is used as a framework for the hypotheses and for interpretation of the results [18]. The model focuses on disability, while the focus in the current study is also on other groups that are underrepresented in the labour market. In the following, the model will be presented, emphasising aspects that are particularly relevant for the current study.

### 1.2 Stone and Colella’s model of factors affecting the treatment of disabled individuals in organizations

Stone and Colella’s model theorize how contextual factors (mainly legislation), organizational factors (i.e. technology, norms, policies) and individual factors (i.e. nature of disability, former contact with people with disabilities, stereotypes) interact to shape psychological assessments and expectations towards people with disabilities. These assessments and expectations are termed “psychological consequences”.

The behaviour that results from these psychological processes elicits certain responses from people with disabilities on the receiving end. Their response to this treatment may then go on to affect contextual, organizational and individual factors. The interactions in the model are recursive, and the model thus manages to capture the malleable nature of the factors included, which may change over the course of time within an organization. This makes the model a well-suited framework for understanding the demand-side factors of employment not only for people with a disability, but for other groups that are under-represented in the labour market. The current study specifically investigates individual factors and psychological consequences of these, as expressed through assessments of job seekers with different characteristics.

While most studies focus on either disability or cultural minorities, the current study investigates willingness to include job seekers representing a range of groups in society that are underrepresented in the workforce, through a vignette study. It also compares the supervisor perspective with the employee perspective, in order to detect organizational level differences when it comes to willingness to include the different job seekers into their work group. Further, the role of experience is explored, and lastly, different types of barriers salient for certain groups are explored. These aims are answered through a mixed methods design, and formulated in three hypotheses and one explorative research question. A normative job seeker described as a single mother is included as one of the vignettes, to function as a reference case.

Hypothesis 1

a: Compared to the single mother vignette, the two characters of a cultural minority will have significantly lower probability of being assessed positively, but will have higher RR of being assessed positively than cases with a mental health or disability issue.
b: Compared to the single mother vignette character, vignette characters with physical disabilities will have significantly lower probability of being assessed positively, but will have higher RR of being assessed positively than vignettes with a mental health issue.
c: Compared to the single mother vignette character, vignette characters with symptoms of mental illness will be least likely to be assessed positively, and will have the lowest RR of being assessed positively.

Hypothesis 2: Supervisors, because of their concern for productivity and absenteeism, will assess vignette characters with a health issue significantly less favourably than employees will.

Hypothesis 3: Respondents who have previous experience with employees similar to the vignette character in question, will assess them significantly more favourably than those who do not have such experience.

Research question: What barriers to including different vignette characters can be identified, and how do these barriers vary between characters?

## 2 Methods

### 2.1 Participants

Respondents (*n* = 1230) were supervisors and middle level managers (*n* = 305), and employees without supervisor responsibilities (*n* = 925). Fifty-three percent were female (*n* = 1207), and mean age was 44 years (*n* = 1180; SD 12.43). In the supervisor subgroup 45%were female (*n* = 300), and mean age was 46 years (*n* = 297; SD 10.22). In the employee subgroup 55%were female (*n* = 907), and mean age was 43 years (*n* = 883; SD 13).

Inclusive Workplace Support Centres (IWSC) at the Norwegian Labour and Welfare Administration in eight Norwegian counties assisted with recruitment, by providing lists of companies to invite for participation. IWSCs are resource centres located in all 18 counties, and their main task is to support workplaces in creating a more inclusive work life. The survey was distributed directly through emails to the supervisors and employees in the companies that agreed to participate. Response rates were 29%among supervisors and 19%among employees.

### 2.2 Instruments

Questionnaires were sent to supervisors and to employees without supervisor responsibilities. The questionnaire included demographic background variables and the Workplace Inclusion Questionnaire (WIQ) [19]. WIQ contains vignettes describing job seekers who differ on certain characteristics, such as cultural background, health, or disability issues. The number and types of vignettes included in a given study may vary depending on the study purpose [19]. The descriptions of health issues are based on formal diagnostic criteria in the ICD-10 and medical encyclopaedias. For vignette characters with a mental illness, the diagnosis was not stated explicitly in order to avoid labelling. Only symptoms were described. The vignettes and questions are available as supplementary materials. In order to reduce the time spent answering the survey, the supervisors were randomly assigned to respond to one of two blocks of vignettes (see Table 1). Employees without supervisor responsibilities were presented with all ten vignettes. The vignette character of the single mother did not describe health issues or a cultural minority, and was included in both blocks. The purpose of this character was to include a more or less normative job seeker with no serious health or disability issues, or other traits known to be related to discrimination in the recruitment process.

**Table 1:** Two blocks of vignettes, randomly assigned to supervisors (employees received all vignettes). Within each block, the vignettes were displayed randomly. M = male F = female

Block 1	Block 2
Single mother (F)	Single mother (F)
Newly arrived immigrant (M)	2nd generation immigrant (M)
Audio impairment (M)	Visual impairment (F)
Wheelchair (F)	Somatization disorder (F)
Depression (F)	Schizophrenic symptoms (M)
ADHD (F)

The vignettes in the questionnaire attempt to give a credible description of an individual with a mental illness, a physical disability, or with a cultural minority background. All vignette characters were approximately the same age, and it was stated that they had the necessary qualifications for the job. Gender varied, with six of the characters being female. Each vignette was followed by the question: “Given the current circumstances, how do you think [name of vignette character] fits into your work group?” The respondent was asked to indicate how well the vignette character in question would fit on a scale from 1 (very poorly) to 5 (very well). If the respondent rated the vignette character negatively or neutrally (1–3 on the scale), a follow-up question was asked: “If [name of vignette character] does not fit quite/very well into your work group: What is the main reason?” Respondents were then asked to indicate their main reason from a set of pre-defined options, such as accommodation, economic consequences, interaction with others, or to fill in an open-ended response. Lastly, respondents were asked to indicate whether or not they had previous experience with an employee or colleague like the vignette character in question (yes/no).

### 2.3 Procedure

The online survey platform Qualtrics was used to collect the data, through distributing emails with a link to the survey. A reminder was sent 1–2 weeks after the initial email. The survey was anonymous and did not store IP addresses. The survey took 10–15 minutes to complete. The survey was part of a larger study, which was submitted to the Norwegian Centre for Research Data (NSD) for consideration. Since no directly or indirectly identifiable data was collected in the survey, NSD deemed that this part of the study was anonymous and therefore did not require active consent. On these grounds, participation in the study was regarded as consent.

### 2.4 Data analyses

The analyses were conducted with SPSS 25 and Excel. Assessments of vignette characters were recoded into dichotomous variables, so that explicitly positive assessments (the character in question fit “Quite well” or “Very well”) were distinguished from neutral and negative assessments (the character in question fit “Neither poorly nor well”, “Quite poorly”, or “Very poorly”).

Frequency analyses showed the distribution of responses in each category (positive vs neutral/negative). In a 2×2 table, type of vignette character was treated as exposure (rows), while type of rating was treated as a condition (columns). Risk ratios for being assessed positively was calculated (with 95%CI), using the vignette character describing a single mother as a reference. Chi square tests of independence (with Yates Continuity Correction) were conducted to examine the relationship between experience and type of assessment, and between supervisor status and type of assessment. Qualitative thematic analysis was used to categorize the open-ended responses in the question about barriers [20]. Two of the authors independently categorized the responses, and where they had categorized a response under two different themes, this was discussed until consensus was reached. Inter-rater reliability before consensus discussions ranged from 59%–84%for the vignette characters.

## 3 Results

The anticipated pattern described in hypotheses 1a–c was only partially supported, as displayed in Table 2. Hypothesis 1a was not supported, as both of the cultural minorities were more, not less, likely to be assessed positively when compared to the single mother character. For the newly arrived immigrant, the difference was marginal (RR 1.10 [1.01, 1.19]), but for the second generation immigrant RR was rather high (RR 1.86 [1.75, 1.97]). Contrary to the assumptions in hypothesis 1b, the character describing audio impairment were slightly more likely to be assessed positively compared to the single mother (RR 1.37 [1.27, 1.47]). The other characters with a physical disability were however less likely to receive a positive assessment. Thus, the assumption in hypothesis 1b, that vignettes with a physical disability would be less likely to be rated positively than the single mother, was not consistently supported. Hypothesis 1c, that vignettes describing a mental illness would be less likely to be positively assessed, and have the lowest RR values was generally supported, but the vignette describing visual impairment was least likely to be rated positively (RR 0.33 [0.29, 0.39]). Apart from the characters with audio and visual impairment, vignette characters with a health or disability issue were generally less likely to receive a positive rating compared to the reference character.

**Table 2:** Risk ratio for receiving positive assessments when compared to the single mother vignette character. RR, *n*, %assessing positively and negatively, and 95%CI

Vignette character	*n*	Positive	*n*	Negative or neutral	*n*	RR	95%CI Low	95%CI High
Newly arrived immigrant	950	56 %	532	44 %	418	1.10	1.01	1.19
Audio impairment	938	70 %	655	30 %	283	1.37	1.27	1.47
Wheelchair user	928	40 %	368	60 %	560	0.78	0.70	0.86
Depression	910	31 %	281	69 %	629	0.60	0.54	0.68
ADHD	903	22 %	199	78 %	704	0.43	0.38	0.49
2nd generation immigrant	896	95 %	851	5 %	45	1.86	1.75	1.97
Visual impairment	891	17 %	152	83 %	739	0.33	0.29	0.39
Somatization disorder	885	27 %	235	73 %	650	0.52	0.46	0.58
Schizophrenic symptoms	882	22 %	190	78 %	692	0.42	0.37	0.48
*Single mother*	*1074*	*51 %*	*549*	*49 %*	*525*

Hypothesis 2, that supervisors would rate vignette characters with a health issue less favourably than employees would, was partially confirmed, as displayed in Table 3. However, effect sizes as demonstrated by the phi coefficient value are low, which indicates that the practical significance of this difference is uncertain.

**Table 3:** Association between role (supervisor vs employee) and assessments of the vignette characters. Degrees of freedom, *n*, %of positive assessments who were supervisors, %of negative/neutral assessments who were supervisors, X^2^ value (Yates Continuity Correction), *p* value and *phi* value

Character	df	*n*	Positive	Negative or neutral	X^2^	*p*	phi
Newly arrived immigrant	1	950	11 %	9 %	0.52	0.472	–0.03
Audio impairment	1	938	9 %	14 %	5.38	0.020	0.08
Wheelchair user	1	928	6 %	13 %	9.84	0.002	0.11
Depression	1	910	6 %	12 %	6.16	0.013	0.09
ADHD	1	903	12 %	10 %	0.22	0.639	–0.02
2nd generation immigrant	1	896	11 %	11 %	0.00	1	0.01
Visual impairment	1	891	10 %	11 %	0.02	0.877	0.01
Somatization disorder	1	885	5 %	13 %	9.48	0.002	0.11
Schizophrenic symptoms	1	882	8 %	11 %	1.59	0.207	0.05
Single mother	1	1074	16 %	19 %	0.96	0.327	0.03

Hypothesis 3, that previous experience would be associated with a more favourable rating of the vignette character in question, was supported across all vignette characters, as shown in Table 4. Effect sizes are small for all vignettes, except for the character using a wheelchair, for which effect sizes are moderate χ^2^ (1, *n* = 910) = 103.20, *p* = 0.000 phi = –0.34). As Table 4 shows, the share of respondents who had previous experience with colleagues similar to the vignette characters varied widely. Very few respondents had previous experience with colleagues with schizophrenic symptoms (*n* = 848, 12%) and visual impairment (*n* = 852, 8%).

**Table 4:** Associations between previous experience with a colleague similar to the vignette character in question, and the assessment of that character. Degrees of freedom, *n*, %of respondents assessing the job seeker positively and having previous experience, %of respondents assessing the job seeker negatively/neutrally and having previous experience, X^2^ (Yates Continuity Correction), *p* value, and *phi* value

Character	df	*n*	Positive	Negative or neutral	X^2^	*p*	phi
Newly arrived immigrant	1	935	62 %	53 %	7.17	0.007	–0.09
Audio impairment	1	929	51 %	27 %	42.54	0.000	–0.22
Wheelchair user	1	910	33 %	7 %	6.16	0.013	–0.34
Depression	1	885	63 %	48 %	15.15	0.000	–0.13
ADHD	1	872	63 %	46 %	16.70	0.000	–0.14
2nd generation immigrant	1	894	58 %	34 %	8.99	0.003	–0.11
Visual impairment	1	852	20 %	6 %	31.79	0.000	–0.20
Somatization disorder	1	833	52 %	44 %	3.96	0.047	–0.07
Schizophrenic symptoms	1	848	20 %	10 %	11.94	0.001	–0.12
Single mother	1	1050	86 %	66 %	58.81	0.000	–0.24

**Fig. 1:**
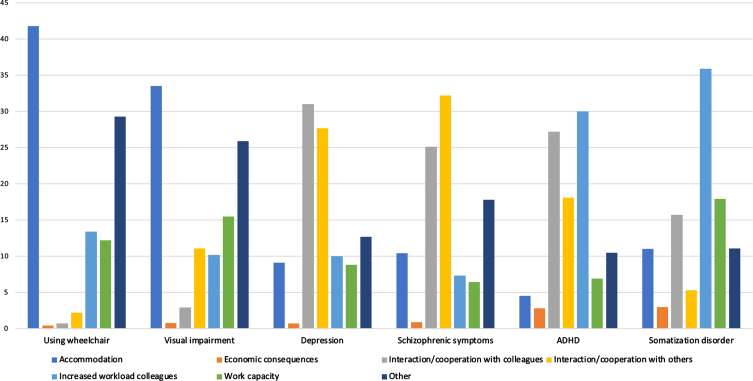
Reasons provided for assessing a case negatively or neutrally, supervisors and employees.

The main reasons for the reluctance towards including the vignette characters with an RR below 1 are presented in Fig. 1. The figure shows a “barrier profile” for each vignette character, with different reasons being emphasized for being reluctant to work with that particular vignette character.

For the vignette characters describing depression, somatization disorder and ADHD, open-ended answers constituted 10–13%of the responses, but for the vignette characters describing schizophrenic symptoms, visual impairment, and using a wheelchair, open-ended responses constituted as much as 18–30%of the responses, which warranted further examination. Table 5 shows the themes that were identified in the open-ended responses, and how many percent of the responses to each vignette character were categorized under each theme. Note that data on barriers was only collected for vignette characters receiving neutral to negative ratings.

**Table 5:** Coding of qualitative themes in open-ended responses, in percent per case

Theme	Using wheelchair (*n* = 123)	Visually impaired (*n* = 132)	Schizophrenic symptoms (*n* = 76)	Depression (*n* = 59)	Somatization disorder (*n* = 41)	ADHD (*n* = 54)
Accommodation	25%	2%	0%	0%	0%	0%
Assumptions about accommodation	17%	14%	0%	2%	2%	0%
Person-specific	5%	4%	29%	36%	27%	11%
Nature of the work	40%	55%	5%	0%	5%	2%
Clients/customers	3%	13%	53%	29%	10%	6%
Sees possibilities	6%	5%	5%	5%	7%	4%
Security	1%	5%	1%	2%	0%	6%
Absenteeism and work environment	0%	0%	1%	14%	29%	11%
Work capacity and production loss	0%	1%	0%	8%	7%	56%
Other	3%	2%	5%	5%	12%	6%
*Inter-rater reliability*	*72%*	*84%*	*76%*	*63%*	*68%*	*59%*

The open-ended response category was most frequently used for the vignette characters with either a visual impairment (*n* = 132) or using a wheelchair (*n* = 123). By comparison, open-ended responses to the other vignette characters counted between 41 and 76. The most cited free-text theme for the character using a wheelchair (49%) and for the character with visual impairment (72%) was “Nature of the work”. Examples of this theme are “Selling products with visual details will be challenging” (visual impairment); and “We do manual labour” (using wheelchair). Examples of statements categorized as “Assumptions about accommodation” are “The work requires travelling throughout the county” (using wheelchair) and “This is a job with phone support” (visual impairment).

For schizophrenic symptoms, the most frequently cited theme in the open response category (40%) consisted of concerns regarding clients or customers, such as children, patients, or business partners, illustrated by quotes such as “Because of customer relations, we can’t have mentally unstable persons” and “Can’t work as a teacher with those kinds of problems.” Moreover, person-specific statements were relatively prevalent (22%): “A danger to himself and others” and “Difficult to relate to” are examples of this.

Nature of the work or issues regarding accommodation are by far the most important free-text themes for the vignette characters with physical disabilities, while person-specific statements and concerns regarding customer care were most frequently cited for schizophrenic symptoms and depression. For somatization disorder concerns with absenteeism and work-environment were most prevalent, followed by person-specific statements. For ADHD, the most cited concerns were work capacity and production loss.

## 4 Discussion

The aim of the study was to investigate employees’ and employers’ willingness to include job candidates with a mental illness, a physical disability, or minority background into their workplace, and exploring factors relating to these assessments. The first hypothesis was only partially supported: Cultural minorities we *more* likely, not less, to be assessed positively when compared to the reference character. Most vignette characters with a physical disability and all characters with a mental health issue were, however, less likely than the reference character to be assessed positively, with the exception of the character with audio impairment. RR values for vignettes with a physical disability were not consistently lower than for mental illnesses, as assumed. Supervisors assessed the vignette characters describing somatization disorder, depression, audio impairment and using a wheelchair less positive than employees without supervisor status. Moreover, respondents who reported to have previous experience with someone similar to the vignette character in question assessed this character more positively than respondents without this experience. Barriers for inclusion varied between vignette characters, however there were some similarities between characters with mental illness, and between characters with a physical disability. The vignette characters describing schizophrenic symptoms, visual impairment, and using a wheelchair elicited the largest share of free-text responses when asked about the reason for not assessing the character positively.

### 4.1 Assessments of the vignette characters

The anticipated pattern of assessments of the different characters was only partially confirmed. The cultural minority characters were assessed positively, and especially the second-generation immigrant. This can be due to social desirability, but it can also be an expression of preferences for job seekers without health issues. Another interesting finding was that the assessments of vignette characters with a physical disability varied widely. This may indicate that respondents made more individualized assessments of these characters than the vignette characters representing mental illnesses. A recent study among employers found that mental health conditions were generally regarded as more diffuse and challenging to handle than physical disabilities, due to the invisibility and expected instability of mental health conditions [11]. This may explain the generally negative assessments of vignette characters with a mental illness in the current study. The share of respondents who had previous experience with colleagues similar to the one in question varied widely, which is likely to explain the lack of positive assessment of particularly the characters with visual impairment and schizophrenic symptoms.

Significant associations were found between supervisor status and assessments of some of the characters. The vignettes describing somatization disorder, depression, using a wheelchair, and having an audio impairment received significantly less positive assessments from leaders compared to employers. As effect sizes were small, the practical significance of these differences is uncertain. Generally, supervisors and employees agreed in their assessments of the characters. This coherence between organizational levels may facilitate inclusion efforts, as the same types of concerns need to be addressed across organisational levels.

Our findings indicate that previous experience with similar colleagues leads to a more positive assessment of the included vignette characters. This finding aligns well with social psychological theories explaining how humans tend to categorize others into ingroups and outgroups, and how prejudices can be reduced through positive interaction [21–23].

Six vignette characters had RR values below 1 of being positively assessed. This should be interpreted in light of the role of experience, as discussed above, as well as respondents’ own explanations for their assessments, which will be discussed in the following.

### 4.2 Reasoning behind assessments

The reasons provided for assessing a vignette character negatively or neutrally give valuable insight into the demand-side barriers for employment for these specific groups, as shown in Fig. 1. For the vignette characters expressing schizophrenic symptoms and depression symptoms, social interaction seemed to be the main concern, while for both somatization disorder and ADHD, increased workload for colleagues was the most frequently cited concern. For both visual impairment and using a wheelchair, accommodation was by far the most frequently cited barrier. The open-ended response category “Other” enabled respondents to provide a free-text response if the predefined categories were not sufficient. This response category provided useful insight into the knowledge and assumptions of the respondents regarding the conditions described. For the vignette character using a wheelchair, “Other” constituted as much as 29%of the responses to the follow-up question. For the vignette characters with visual impairment and with schizophrenic symptoms, this category constituted 26%and 18%of the responses, respectively.

### 4.3 Analysis of open-ended responses

The pre-defined categories were identified through discussions with supervisors during the development of the questionnaire. Even so, the free-text responses provide even more ecologically valid responses than the pre-defined categories, as the respondents were able to provide unique insights into how organizational characteristics, nature of the job, or attributes of the vignette character in question shaped their assessment. Many of the free-text responses regarding the job seeker with a visual impairment or using a wheelchair were coded into themes already available from or similar to the pre-defined categories, perhaps indicating a need to rationalize one’s negative assessment of these particular characters. Some of the quotes presented in the results, that were categorized under the theme “Accommodation” or “Assumptions about accommodation”, indicate lack of knowledge about the existence and quality of different aids that are available to people with different types of physical disabilities.

The findings in the current study are in line with the theorized relationships made in Stone and Collella’s model of factors influencing the treatment of people with disabilities in an organization [18]. Results support that characteristics of the observer, of the job seeker, and of the workplace contribute to shape how people with disabilities or health issues are assessed. In the model, “nature of the job” is assumed to interact with individual factors on the part of both the observer and the person with a disability, which in turn affect how observers treat someone with a disability [18]. Although these specific relationships were not tested statistically in the current study, these aspects are prevalent in respondents’ own reasoning when explaining the rationale behind the assessment of the vignette characters. A relevant point in this regard made by Stone and Collella, is that supervisors are likely to picture an idealized or customary way of performing a certain job, while at the same time consider the work ability of a disabled person based on more or less faulty assumptions [18]. This creates an overestimated gap between essential job requirements on the one hand, and a disabled person’s actual ability to perform that job on the other hand. The findings in the current study can to some degree be interpreted to support this notion, as lack of knowledge on both technical aids and the work ability of the different vignette characters were evident in the respondents’ reasoning.

### 4.4 Implications of the findings

The findings are somewhat similar to what has been found in other studies about willingness to include and accommodate job seekers with disabilities or health issues [24–26]. All in all, the barriers indicated for the different vignette characters seem to represent a mix of experiences, workplace-specific circumstances, lack of knowledge, and poor attitudes. However, an important contribution of the current study is that it shows what type of barriers are pertinent to which type of health issue or characteristic. The analysis of the open-ended responses gives insight into respondents’ assumptions about work ability, especially for the vignette characters describing visual impairment and using a wheelchair, where open-ended responses were most prevalent. The findings are particularly useful for vocational rehabilitation service providers, which can play an important role as an intermediary between the job seeker and the workplace [27, 28]. Many companies have diversity statements and policies, however, this is not always associated with actually recruiting people with diverse backgrounds [29], implicating that insecurity and/or stigma held by supervisors –or employees –still negatively affect hiring processes. Studies have found that although employers express willingness to hire diverse applicants, many are unsure where to start, and want closer co-operation with vocational rehabilitation agencies [26, 30]. The need for an intermediary link, such as an employment specialist, between employers and job seekers is made clear both in the current study, as well as in previous studies [26, 31–33]. Something as simple as increasing knowledge about accommodation possibilities may create more positive attitudes [16]. The responses provided in the open-ended response category indicates that this kind of knowledge is indeed still lacking among supervisors and employees. Vocational rehabilitation agencies can help bridge this knowledge gap regarding accommodation possibilities, technical aids, reimbursement of accommodation costs, and perhaps most importantly: To provide a job match between employers’ actual needs and job seekers’ competence and motivation [28, 34, 35]. Focusing on job match and follow-along supports for both the job seeker and the employer may help mitigate the barriers that have been identified in the current study.

### 4.5 Implications for research

Future studies could add measures of contextual factors, and address for example perceived organizational culture and its relationship with assessment of job seekers like the ones used in this study. Moreover, longitudinal designs are rare among studies investigating employers’ perspective on diverse recruitment. Future studies can measure how perceptions of different job seekers develop over time within the same industries, and see this in relation to actual hiring practices during that same period of time.

### 4.6 Strengths and limitations

By using a vignette design instead of simply listing diagnoses, condition or cultural background, we attempted to facilitate identification while avoiding labelling based on stereotypes and lack of knowledge about specific diagnoses. We assumed that providing information about behaviour and symptoms common to a condition might increase the ecological validity. A few limitations should be pointed out, however. First, neither the vignettes themselves nor the responses they elicit, take specific contextual factors into consideration, such as cross-pressures or external demands affecting the respondent. There is a risk of eliciting responses to a hypothetical situation that differs from real-life decisions [36]. Furthermore, vignettes such as the ones used in this study do not adequately cover the diversity of how a certain diagnosis or disability manifests. All the persons described in the vignettes are in their 30’s and 40’s, which make them more comparable to each other, but not representative for the broader workforce population. The vignette characters describing mental illness did, however, conform to symptoms fulfilling each of the relevant diagnostic criteria, allowing complex health conditions to be described clearly without stating the diagnosis explicitly, while keeping information about age and qualifications constant. Meanwhile, the gender of the vignette characters was random, and we suggest that future studies manipulate the gender of the vignette characters to control for potential gender effects. This would have required a longer questionnaire, and since gender was not the topic under study, it was not included as an experimental variable. Response rate was low, but some factors may compensate for this: The sample size is relatively large, a broad range of industries is represented, both the supervisor and the employee perspectives are included, and there is gender balance in the sample. Finally, when measuring normative phenomena, such as liking or attitudes towards certain people, socially desirable answers become particularly likely. Hence, although the patterns found in the current study may indeed reflect common attitudes, they might in reality be even more pronounced.

## 5 Conclusion

This study is unique in the sense that it broadly investigates supervisors’ and employees’ assessments of a range of disfavoured groups in the workforce, instead of focusing on only one or a few target groups. Its main contribution is to enable a comparison of the status quo of how employers and employees assess groups who are underrepresented in the work force, as well as give an account of the reasoning behind the reluctance to include individuals with certain mental health or disability issues.

The findings of the study indicate that both supervisors and employees are generally reluctant to include job seekers who have a mental illness or physical disability as compared to a job seeker with no serious health or disability issues. Supervisors and employees are generally concurrent in their willingness or reluctance to include the different vignette characters, and previous experience is associated with more favourable ratings for all vignette characters. The findings are useful for practitioners working with vocational rehabilitation, as it shows what types of barriers supervisors and employees perceive when considering job seekers with different types of mental illness or physical disabilities. Increasing knowledge about accommodations and public funding for these, as well as providing on-the-job-supports for both the job seeker and the employer can help overcoming barriers and increase work participation among underrepresented groups in the workforce. For policy makers, the findings underline that employers need support and information in order to actively recruit employees from underrepresented groups.

## Data storage and availability

The data that support the findings of this study, as well as the questionnaire, are available from http://dx.doi.org/10.18712/NSD-NSD2769-V1.
